# Identification of the novel 3′ UTR sequences of human IL-21 mRNA as potential targets of miRNAs

**DOI:** 10.1038/s41598-017-07853-x

**Published:** 2017-08-10

**Authors:** Yutaka Enomoto, Rie Takagi, Yutaka Naito, Tsuyoshi Kiniwa, Yasuhito Tanaka, Susumu Hamada-Tsutsumi, Masaaki Kawano, Sho Matsushita, Takahiro Ochiya, Atsushi Miyajima

**Affiliations:** 1 Laboratory of Cell Growth and Differentiation, Institute of Molecular and Cellular Biosciences, The University of Tokyo, 1-1-1 Yayoi, Bunkyo-ku, Tokyo, 113-0032 Japan; 2 Department of Allergy and Immunology Faculty of Medicine, Saitama Medical University, 38 Morohongo, Moroyama-cho, Iruma-gun, Saitama, 350-0495 Japan; 3 Division of Molecular and Cellular Medicine, National Cancer Center Research Institute, 5-1-1 Tsukiji, Chuo-ku, Tokyo, 104-0045 Japan; 4 Department of Virology and Liver Unit, Nagoya City University Graduate School of Medical Sciences, 1 Kawasumi, Mizuho-ku, Nagoya, 467-8601 Japan

## Abstract

Hepatitis B virus (HBV) infection is a leading cause of hepatocellular carcinoma worldwide. However, the strategy of HBV to escape from the host immune system remains largely unknown. In this study, we examined extracellular vesicles (EVs) secreted from human hepatocytes infected with HBV. EVs includeing exosomes are nano-size vesicles with proteins, mRNAs, and microRNAs (miRNAs), which can be transmitted to different cells. We found that 104 EV associated miRNAs were increased in hepatocytes more than 2-fold by HBV infection. We then selected those that were potentially implicated in immune regulation. Among them, five HBV-induced miRNAs were found to potentially target multiple sequences in the 3′UTR of IL-21, a cytokine that induces anti-viral immunity. Moreover, expression of a reporter gene with the 3′ UTR of human IL-21 mRNA was suppressed by the five miRNAs individually. Finally, IL-21 expression in cloned human T cells was down-regulated by the five miRNAs. Collectively, this study identified the novel 3′ UTR sequences of human IL-21 mRNA and potential binding sites of HBV-induced EV-miRNAs.

## Introduction

Extracellular vesicles (EVs) including exosomes are nano-size membrane vesicles released from many cell types and contain various cellular components, including proteins, mRNAs, and microRNAs (miRNAs)^1, 2^. As EVs from one cell can be transferred to another cell, they play a role of mediators for intercellular communication^1–3^. EVs are found in diverse body fluids such as blood, milk, urine, and saliva and have been implicated in diverse biological functions and diseases^4–7^. For example, tumor-derived EVs can induce numerous immune suppressive pathways such as apoptotic signaling, immune suppressive activity, and blockade of receptors/ligands involved in anti-cancer immunity, promoting tumorigenesis, angiogenesis, and cancer metastasis^8–15^. EVs released from cells infected with various types of viruses exhibit functions that promote infection and propagation of viruses by suppressing host immunity and modification of their microenvironment^16, 17^. EV associated miRNA (EV-miRNA) profiles in sera are different between patients and healthy donors and reflect the physiological and pathological status^18, 19^, while the mechanism underlying the formation of EVs remains largely unknown. As a sensitive means to analyze miRNA has been developed, serum EV-miRNA profiles provide useful diagnostic biomarkers for prediction of disease progression.

Hepatitis B virus (HBV) infection is a leading cause of hepatocellular carcinoma (HCC) worldwide. Since HBV is a noncytopathic DNA virus, inflammation in the liver is mediated by host immune responses to the HBV-infected hepatocytes^20, 21^. HBV infection in adults results in a transient liver disease and the virus is cleared in more than 95% of adults, whereas more than 90% of neonates exposed to HBV at birth become persistently infected, suggesting that HBV needs to escape from the host immune system for its persistent infection^22, 23^. Recent studies on EV-miRNAs from patients infected with HBV have revealed that the profiles of serum EV-miRNAs are altered by disease progression^24, 25^. However, the function of each EV-miRNA detected in HBV patients remains largely unknown.

In this study, we investigated EV-miRNAs secreted from HBV-infected human hepatocytes and found that various cytokines involved in anti-viral immunity could be targeted by EV-miRNAs secreted from HBV-infected hepatocytes. We found that five miRNAs were up-regulated in HBV-infected hepatocytes, which potentially target the sequences of the 3′UTR of human IL-21 mRNA and down-regulated IL-21 mRNA expression in human T cells. As IL-21 is known to be an important cytokine involved in anti-virus immunity^26–28^, IL-21 could be a favorable target to escape from the host immune system.

## Results

### EV-miRNA profiles are altered by HBV infection

**Figure 1:**
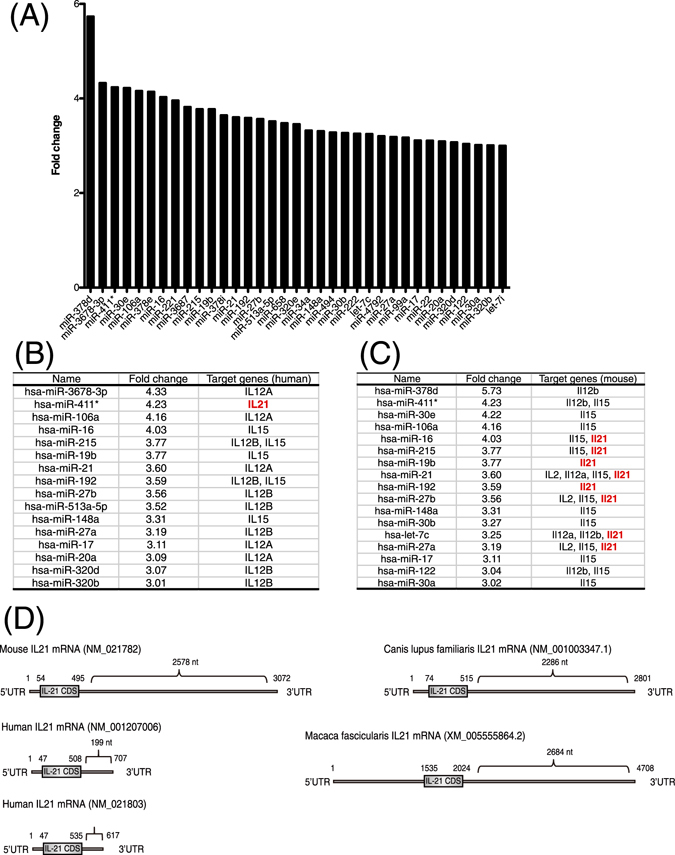
EV-miRNAs secreted from human hepatocytes infected with HBV. (**A**) EV-miRNAs secreted from human hapetocytes with HBV-infection or Non-infection were analyzed by performing the microarray. EV-miRNAs with more than 3-fold upregulated by HBV-infection are plotted. (**B**) Human candidate target genes of EV-miRNAs with more than 3-fold upregulated by HBV-infection are listed. (**C**) Murine candidate target genes of EV-miRNAs with more than 3-fold upregulated by HBV-infection are listed. (**D**) The 3′ UTR of mouse, human, Canis lupus familiaris, and Macaca fascicularis IL-21 mRNA registered in GenBank are illustrated. Nucleotide numbers are shown.

To investigate EV-miRNAs secreted from human hepatocytes infected with HBV, we utilized PXB chimeric mice, in which liver is highly repopulated with human hepatocytes. We infected hepatocytes from PXB-mice with HBV. The HBV infection was confirmed by measuring extracellular HBV DNA (Fig. S1). At 5th day after the infection, we harvested EVs from the culture media and performed microarray analysis of the EV-miRNAs. We found that 104 EV-miRNAs were up-regulated more than 2-fold by HBV infection (Table S1). In order to find the EV-miRNAs that may affect the host anti-viral immunity, we performed a bioinformatics search using Targetscan 6.2 (http://www.targetscan.org/vert_61/). Interestingly, a large number of EV-miRNAs up-regulated more than 3-fold by HBV were found to potentially target several anti-viral cytokines such as IL-12p35, IL-12p40, IL-15, and IL-21 (Fig. 1A,B). Since gene regulatory mechanisms by miRNAs are well conserved among different animal species, we also analyzed target genes from murine transcripts and found that many EV-miRNAs could target several anti-viral cytokines (Fig. 1C). Curiously, however, eight EV-miRNAs were found to potentially target murine IL-21, whereas only one miRNA was found for human IL-21 (Fig. 1B,C). We then compared human IL-21 mRNA with murine IL-21 mRNA in databases and found that the length of 3′UTR of human IL-21 mRNA registered in various databases (GenBank, Ensembl, and UCSC Genome Browser) was much shorter than that of murine IL-21 mRNA (Fig. 1D). Moreover, long 3′UTR of IL-21 mRNA of other species such as Canis lupus familiaris and Macaca fascicularis are also registered in databases (Fig. 1D).

### Identification and characterization of the 3′ UTR of human IL-21 mRNA

**Figure 2:**
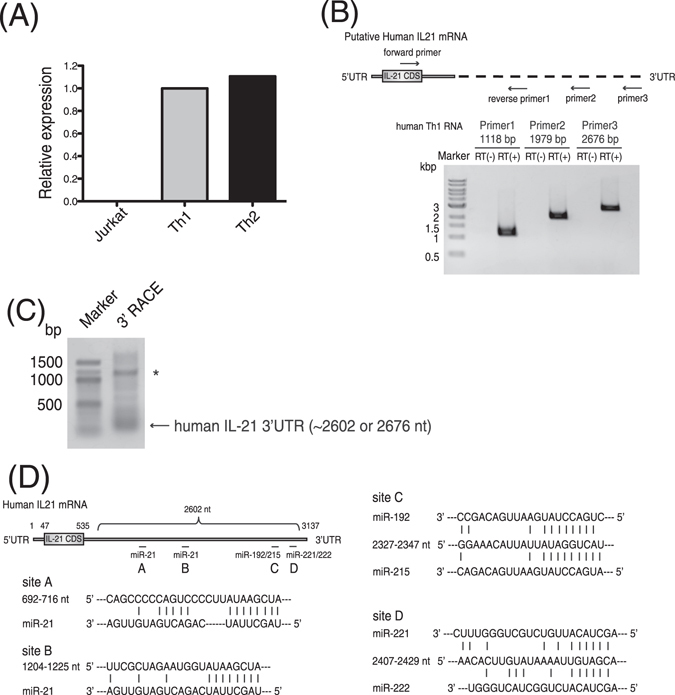
Long 3′ UTR of human IL-21 mRNA was identified from human T cells. (**A**) Relative expression level of IL-21 mRNA was examined by quantitative real-time RT-PCR. Data shown are a representative of three independent experiments performed. Bars indicate the means of triplicates. (**B**) RT-PCR was performed with RNA from human Th1 cells to detect human IL-21 mRNA. Revere primers were designed in putative regions of 3′ UTR of human IL-21 mRNA. One representative data of three experiments is shown. (**C**) The 3′ end of human IL-21 mRNA was identified by the 3′ RACE experiment. The forward primer was designed to anneal to the 2463–2484 nt of the 3′ UTR. Asterisk indicates a non-specific band. (**D**) The identified 3′ UTR of human IL-21 mRNA and its conserved target sites of the EV-miRNAs are illustrated.

IL-21 is known to control chronic viral infection^26–28^ and has been shown to play a pivotal role for immune responses in a mouse model of HBV infection^23^. In addition, several clinical studies reported the importance of IL-21 expression for inducing anti-viral immunity and anti-viral therapy against HBV infection^29–33^. Thus, we hypothesized that HBV might escape from the host immune system by down-regulating IL-21 expression. However, the 3′UTR of human IL-21 mRNA in the multiple databases were significantly shorter than mouse IL-21 mRNA and the regulation of IL-21 expression through its 3′UTR has not been reported previously. Since the major source of IL-21 is T lymphocyte^34^, we first examined which types of human T cells express IL-21 and revealed that IL-21 was highly expressed in primary Th1 and Th2 cells derived from human PBMC, whereas its expression in Jurkat T lymphoma cells was hardly detected (Fig. 2A). Next, we investigated whether a longer 3′UTR of human IL-21 mRNA is present by PCR analysis of human Th1 cells mRNA. We designed the forward oligonucleotide primer at coding sequences (CDS) of the mRNA and reverse oligonucleotide primers at putative 3′UTR of the mRNA based on the genomic DNA sequence. We then found that long cDNAs derived from the 3′UTR of human IL-21 mRNA were amplified from cDNAs of human Th1 cells (Fig. 2B). Next, we identified the end sequences of human IL-21 mRNA by 3′ RACE analysis to be 2602 or 2676 nucleotides from the start of the 3′UTR (Fig. 2C). The long cDNAs of 3′UTR of human IL-21 mRNA were also amplified from cDNAs of human Th2 cells derived from an independent healthy donor (Fig. S2A,B). To reveal whether or not the human IL-21 mRNA with the long 3′ UTR is a major transcript, we performed quantitative real-time RT-PCR analysis by using two primer pairs: one amplified the CDS region of IL-21 mRNA and the other amplified the terminal region of its 3′ UTR (Fig. S3A,B). We also identified major poly A signal sequences, AAUAAA or AUUAAA^35, 36^, at 1788 nt, 1874 nt, and 2581 nt in the long 3′UTR of human IL-21 mRNA (Table 1 and Fig. S4). Since mRNA 3′-end processing, cleavage and polyadenylation occurr at approximately 20 nt downstream of the poly A signal sequences^35^, AUUAAA at 2581 nt is likely used as a poly A signal in the long 3′UTR of human IL-21 mRNA. AUUAAA is also present at 2557 nt, approximately 20 nt upstream of the 3′-end of the murine IL-21 mRNA (Table 1). These results indicate that the long 3′UTR of IL-21 mRNA are conserved between mouse and human and that it is the major transcript (Fig. S3B). In the long 3′UTR of human IL-21 cDNA we identified, there are multiple conserved miRNA binding sites for miR-21, miR-192, miR-215, miR-221, and miR-222, (Fig. 2D and Fig. S4). These five miRNAs were up-regulated by HBV infection at 2nd and 5th day after the infection (Tables S1 and S2). We also confirmed that the expression levels of these miRNAs were increased by quantitative real-time RT-PCR (Fig. S5).

**Table 1:** Polyadenylation (poly A) sites in the 3′ UTR of human or mouse IL21.

poly A signal	AAUAAA	AUUAAA
hIL-21 3′ UTR (2602 nt)	1874 nt	1788 nt 2581 nt
mIL-21 3′ UTR (2578 nt)	2389 nt	2557 nt

### HBV-induced EV-miRNAs reduce the expression of human IL-21 through its 3′UTR

**Figure 3:**
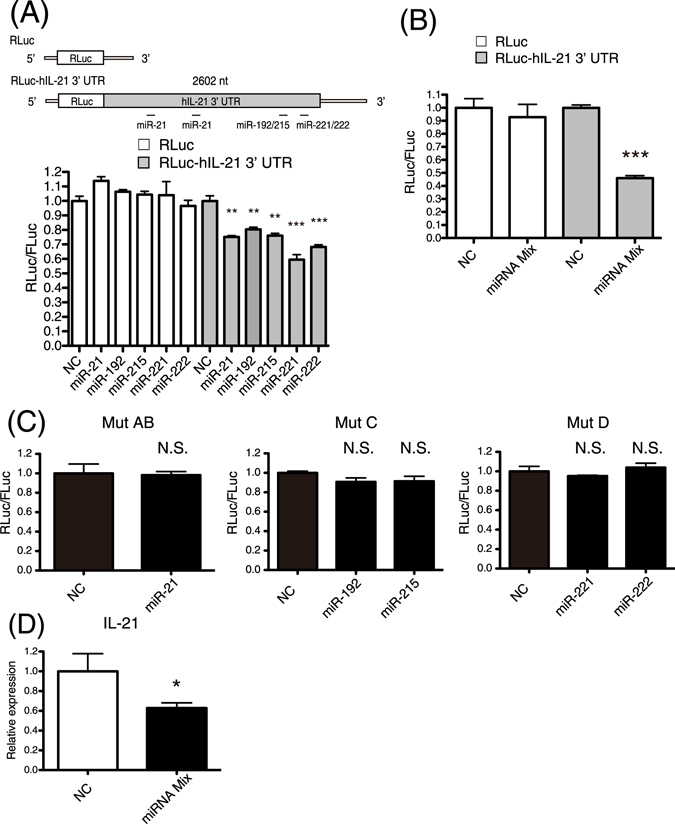
The expression of human IL-21 was suppressed by miRNAs. (**A**) 293 T cells were co-transfected with a reporter vector (Renilla or Renilla-hIL-21 3′ UTR), a transfection control vector (pGL3-control), and negative control RNA (NC) or miRNA. Final concentration of NC or miRNAs was 100 nM. Luciferase assays were performed at 24 hours after the transfection. (**B**) Luciferase assays were performed as described in (**A**). NC (final 250 nM) or mixture of miR-21, 192, 215, 221, and 222 (final 50 nM each) were co-transfected into 293 T cells. (**C**) Luciferase assays were performed as described in (A) with reporter vectors with mutant 3′ UTR of human IL-21. (**D**) NC (final 250 nM) or mixture of miR-21, 192, 215, 221, and 222 (final 50 nM each) were co-transfected into human Th2 cells. Relative expression levels of IL-21 mRNA were examined by quantitative real-time RT-PCR at 24 hours after the transfection. The expression levels were normalized to GAPDH. Data are shown as means + SEM of four independent experiments performed. (**A**–**D**) Data shown are a single experiment representative of three independent experiments performed. *P < 0.05, **P < 0.01, ***P < 0.001. N.S., not significant.

To confirm whether the expression of human IL-21 is suppressed by the five EV-miRNAs, the long 3′ UTR of human IL-21 mRNA was fused to the downstream of the Renilla luciferase (Fig. 3A). Luciferase assays revealed that miR-21, miR-192, miR-215, miR-221, and miR-222 repressed the expression of the reporter gene with the long 3′ UTR of human IL-21 mRNA (Fig. 3A). In addition, the mixture of the five EV-miRNAs efficiently repressed the expression of the reporter gene with the long 3′ UTR of human IL-21 mRNA (Fig. 3B). The expression of reporter gene with mutant 3′ UTR of human IL-21 was not repressed by the five miRNAs, indicating that the five miRNAs directly target human IL-21 gene (Fig. 3C and Fig. S6). We then examined whether the expression level of IL-21 mRNA was repressed in human cloned Th2 cells by transfecting the five miRNAs. Quantitative real-time RT-PCR confirmed that the IL-21 mRNA level in Th2 cells was reduced by transfection of the five miRNAs (Fig. 3D and Fig. S7). In addition, we investigated whether Entecavir, which is an inhibitor of HBV replication, affects the expression of the five EV-miRNAs in HepG2.2.15.7 cells, which are stably transfected with a complete HBV genome. Interestingly, we found that the expression of the five EV-miRNAs were up-regulated by treatment with Entecavir (Fig. S8).

## Discussion

In this study, we have shown that approximately 100 EV-miRNAs were up-regulated in human hepatocytes by HBV infection. In addition, the identification of the long 3′ UTR of human IL-21 mRNA and transfection studies revealed that human IL-21 mRNA with the long 3′ UTR is down-regulated by miR-21, miR-192, miR-215, miR-221, and miR-222, which were in the top 25 miRNAs up-regulated by HBV infection. Previously the expression of miR-21, miR-221, and miR-222 was reported to be increased in EVs from patients infected with HBV^24, 25^ and miR-215 was also shown to increase in the serum derived from patients infected with HBV^37^. Notably, the expression levels of these miRNAs were increased along the progression of disease state. While the expression of miR-192 in patients infected with HBV was not reported, its expression in HepG2.2.15 cells was higher than parental HepG2 cells^38^. In addition, previous reports show that Hepatitis B virus X protein (HBx) induces up-regulation of miR-21 and miR-221^39–41^. Although the mechanism by which the five miRNAs are increased in EVs are unknown, these previous studies indicate that the expression of the five miRNAs is increased by HBV infection.

IL-21 is important for exclusion of HBsAg and production of anti-HBs antibody in a mouse model of HBV infection^23^. Patients acutely infected with HBV express more IL-21 mRNA in their PBMCs compared with healthy individuals^23^. However, the expression level of IL-21 of patients with chronic HBV infection during actively flaring disease and inactive chronic HBV carriers were similar to that of healthy individuals^23^. In addition, IL-21-producing CD4+ T cells contribute to viral control^29^ and high serum IL-21 levels predict HBeAg seroconversion after antiviral therapy in patients with chronic hepatitis B^30^. Therefore, IL-21 could be a favorable target for HBV to escape from the host immune system. In this study, we have shown that the expression of human IL-21 in T cells was repressed by transduction of miR-21, miR-192, miR-215, miR-221, and miR-222. The expression of these five EV-miRNAs were up-regulated by treatment with Entecavir in HepG2.2.15.7 cells. Since Entecavir inhibits HBV replication but does not completely eliminate HBV, HBV might strategically secrete the five EV-miRNAs to survive.

Interestingly, it was reported that miR-21, miR-192, miR-215, miR-221, and miR-222 were enriched in exsosomes derived from sera of cancer patients and supernatants of cancer cells^42–47^. In addition, IL-21 has been shown to exhibit anti-cancer activity as well as anti-viral activity^34, 48^. Therefore, cancer cells might repress the expression of IL-21 via their EVs to create a favorable microenvironment for the cancer cells to proliferate. Collectively, the present study is the first demonstration that human IL-21 mRNA is down-regulated by miR-21, miR-192, miR-215, miR-221, and miR-222, suggesting a novel regulatory mechanism of IL-21 expression in immune responses. However, further studies are necessary to reveal the activities of the HBV-induced EV-miRNAs against human IL-21 mRNA.

## Methods

### HBV infection

Primary human hepatocytes (PHHs) were purchased from PhoenixBio Co., Ltd. (Higashihiroshima, Japan). PHHs were plated on collagen-coated 6-well plates at a density of 2 × 10^6^ cells per well and cultured in dHCGM (Dulbecco’s modified Eagle medium supplemented with 10% fetal bovine serum, 1 μg/mL of penicillin, 1 μg/mL of streptomycin, 20 mM HEPES, 15 μg/mL of L-proline, 0.25 μg/mL of human recombinant insulin, 50 nM dexamethazone, 5 ng/mL of human recombinant epidermal growth factor, 0.1 mM ascorbic acid, and 2% DMSO). PHHs were infected with HBV genotype C-containing serum from human hepatocyte chimeric mice at 25 viral genomes per cell in the presence of 4% polyethylene glycol (PEG) 8000 for 24 hours. The cells were washed three times on day 1 and 2 after the infection to remove the inoculum and cultured in the medium without PEG. The culture supernatants of HBV-infected and uninfected PHHs were collected.

### Extracellular vesicles (EVs) isolation

The medium of HBV-infected PHHs, HepG2.2.15.7 cells, and HepG2 cells was collected and centrifuged at 2000 g for 10 min at 4 °C. The supernatant was filtered with 0.22-μm filter unit (Millipore, USA) to remove cellular debris. Next, the supernatant was ultracentrifuged in Beckman SW41Ti rotor at 35,000 rpm for 70 min at 4 °C. The pellets were washed with 11 mL of PBS (−) and ultracentrifuged again. Finally, the pellets were resuspended in PBS (−).

### Establishment of human allo-reactive T-cell clones by mixed lymphocyte reaction (MLR)

HLA-DR-non-shared PBMC of two donors were suspended in RPMI-1640 medium containing 10% human serum, 1% L-glutamine, 50 IU/ml penicillin, 50 μg/ml streptomycin and were co-cultured (2 × 10^3^/individual donor/well of micro-culture plate 163118, NUNC, Denmark) at 37 °C in a humidified atmosphere with 5% CO_2_, to induce MLR. IL-4 (50 ng/ml; PeproTech, USA) was added in these MLR cultures, to induce Th2 cells. After an 8-day culture, the wells where cells proliferated were typically 5–10% of all the culture wells. The proliferating wells were split into 2 wells of a 96-well flat-bottomed culture plate (Falcon, USA), followed by feeding with irradiated (30 Gy) PBMC used for MLR (1 × 10^5^ / well). After 6 days of culture, the supernatant fluids were harvested to be subjected to cytokine determination by ELISA. After an additional 24-h culture, typical Th1 and Th2 cells were cloned by limiting dilution at 0.3–1.0 T cells/well in the presence of irradiated PBMC. The Th1 and Th2 cell clones which showed marked IL-21 production were used in this study. Informed consents were obtained from healthy volunteers and this study with the peripheral blood of healthy volunteers was approved by the ethics committees of the Saitama Medical University and the University of Tokyo. This study was performed in accordance with Japanese government guidelines.

### Cell culture

293T cells were cultured in DMEM containing 10% FBS and gentamycin. Jurkat cells were cultured in RPMI-1640 medium containing 10% FBS and gentamycin. Human cloned Th2 cells were cultured with RPMI-1640 medium containing 10% FBS. At 6 days after the stimulation with irradiated allogeneic PBMC, human cloned Th2 cells were transfected with NC-RNA (final 250 nM) or mix of miR-21, 192, 215, 221, and 222 (final 50 nM each) (Bioneer, Korea) using Lipofectamine RNAiMAX Transfection Reagent (ThermoFisher, USA). HepG2.2.15.7 cells, subcloned from HepG2.2.15 cell line that are stably transfected with an HBV genome^49, 50^, exhibited high HBV replication levels. HepG2.2.15.7 cells were cultured in DMEM/F12 medium (ThermoFisher) supplemented with 10% fetal bovine serum, 100 U/mL Penicillin, 100 μg/mL Streptomycin, 400 μg/mL Geneticin and 5 μg/mL Insulin. HepG2 cells were cultured in DMEM/F12 medium (ThermoFisher) supplemented with 10% fetal bovine serum, 100 U/mL Penicillin, 100 μg/mL Streptomycin and 5 μg/mL Insulin.

### Entecavir treatment

HepG2.2.15.7 cells were cultured with 1–3 nmol/L Entecavir for 6 day. Before collection of the culture medium, the cells were washed with PBS, and the medium was switched to Advanced DMEM containing 100 U/mL Penicillin, 100 μg/mL Streptomycin and 2 mmol/L L-glutamine. After incubation for 48 hours, the conditioned medium was collected to isolate EVs.

### RT-PCR

Total RNAs were extracted from human T cells with TRIZOL Reagents (ThermoFisher), treated with Deoxyribonuclease I (ThermoFisher), and reverse transcribed by using PrimeScript RT Master Mix (Takara Bio Ink, Japan). Fragments of IL-21 were amplified by using Blend Taq (Toyobo, Japan) with a forward primer 5′-gattcaaatcacttctccaaaag-3′ and reverse primer 1 5′-gcctcttggtttgtctcctg-3′, reverse primer 2 5′-tgttcaagtctcactgcttc-3′, or reverse primer 3 5′-tactgggcgggtagtattta-3′. Relative expression levels of IL-21 mRNA were measured by quantitative real-time RT-PCR performed on a LightCycler 480 (Roche Applied Science, Germany) using SYBR premix Ex Taq reagent (Takara Bio Ink). GAPDH was used as an internal control. The sequence of primers was as follows: 5′-aggaaaccaccttccacaaa-3′ and 5′-gaatcacatgaagggcatgtt-3′ for IL-21 and 5′-agccacatcgctcagacac-3′ and 5′-gcccaatacgaccaaatcc-3′ for GAPDH. For analysis of miRNA expression levels, total RNAs were treated with Deoxyribonuclease I (ThermoFisher) and reverse transcribed by using miScript II RT Kit (QIAGEN, Germany). Mature miRNAs expression was measured using a miScript SYBR Green PCR Kit (QIAGEN). U6 was used as an internal control. Quantitative real-time RT-PCR were performed with a triplicate set. For quantification of EV-miRNAs, total RNAs were isolated from EVs using miRNeasy Mini Kit (Qiagen, Valencia, CA). EVs were diluted with 500 μL of Qiazol solution. After 5 min incubation, 10 μL of 0.1 nM cel-miR-39 were added into each aliquot. Subsewuent phenol extraction and filter cartridge work were carried out according to the manufacture’s protocol. The expression of EV-miRNAs was assessed with quantitative real-time RT-PCR as described previously^3^. PCR was carried out in 96-well plate in StepOne Plus and TaqMan Universal PCR Master Mix (ThermoFisher). All TaqMan MicroRNA assays were purchased from ThermoFisher. Cel-miR-39 was used as an invariant control for EV samples. Detection of EV RNAs was performed by using Bioanalyzer 2100 (Agilent Technologies, Santa Clara, CA). For quantification of HBV DNA, total DNA was extracted from culture media of HBV-infected PHH using SMITEST EX R&D Kit (Genome Science Laboratories, Tokyo, Japan). Extracellular HBV DNA was quantified by real-time quantitative PCR using StepOne Plus and TaqMan Universal PCR Master Mix (ThermoFisher). HBV DNA was amplified using primers HBV-F (5′-CACATCAGGATTCCTAGGACC-3′), HBV-R (5′-AGGTTGGTGAGTGATTGGAG-3′), and TaqMan probe HBV-FT (5′-FAM-CAGAGTCTAGACTCGTGGTGGACTTC-TAMRA-3′).

### 3′-RACE (Rapid Amplification of cDNA Ends)

3′-RACE experiments were performed by using 3′-Full RACE Core Set (Takara Bio Ink) according to the manufacturer’s instructions. The forward primer, 5′-catatgcatctgagaatttagc-3′, was designed to anneal to the 2463–2484 nt of the 3′ UTR of human IL-21.

### Cloning of the full-length 3′ UTR of human IL-21 mRNA

The 3′ UTR of the human IL-21 was PCR amplified from cDNA of human Th1 and Th2 cells by using Phusion High-Fidelity DNA Polymerase (New England Biolabs, USA). The sequence of primers was as follows: 5′-ggatctaacttgcagttgga-3′ and 5′-tactgggcgggtagtattta-3′. PCR fragments were cloned into pCR-Blunt vector (ThermoFisher) and the sequences were identified.

### Luciferase assay

The 3′ UTR was fuesed to downstream of the Renilla luciferase stop codon in pGL4.74 vector (Promega, USA). To generate the mutant 3′ UTR of human IL-21, two-step PCR mutagenesis was performed^51^ using the WT-3′ UTR as a template. 293 T cells cultured in 96-well plate were co-transfected with the reporter vector (Renilla or Renilla-hIL-21 3′ UTR) (2 ng), a transfection control vector expressing firefly luciferase (pGL3-control) (20 ng), and negative control RNA (NC) or miRNA (Bioneer) using Polyethylenimine. Cells were harvested at 24 hours post-transfection and assayed with Dual Luciferase Assay (Promega). Three independent experiments were performed with a triplicate set.

### Statistical analysis

Unless otherwise stated, data are shown as means + SEM and were compared using one-tailed Student’s t test. A value of *P* < 0.05 was taken to indicate statistical significance. Statistical analyses were performed using GraphPad Prism (GraphPad Software, USA).

## Electronic supplementary material

Supplementary information
