# Supplementary material for: Identification of the novel 3′ UTR sequences of human IL-21 mRNA as potential targets of miRNAs
Source: Sci Rep. 2017 Aug 10;7:7780. doi: 10.1038/s41598-017-07853-x (PMC5552845; doi:10.1038/s41598-017-07853-x)
Supplement: Supplementary file 1 — Supplementary information [file 41598_2017_7853_MOESM1_ESM.pdf]

Supplementary information

Identification of the novel 3' UTR sequences of human IL-21 mRNA as potential targets of miRNAs

Yutaka Enomoto[1,*], Rie Takagi[2,$], Yutaka Naito[3,$], Tsuyoshi Kiniwa[1], Yasuhito Tanaka[4], Susumu Hamada-Tsutsumi[4], Masaaki Kawano[2], Sho Matsushita[2], Takahiro Ochiya[3], Atsushi Miyajima[1,*]

[1]Laboratory of Cell Growth and Differentiation, Institute of Molecular and Cellular Biosciences, The University of Tokyo, 1-1-1 Yayoi, Bunkyo-ku, Tokyo, 113-0032, Japan

[2]Department of Allergy and Immunology Faculty of Medicine, Saitama Medical University, 38 Morohongo, Moroyama-cho, Iruma-gun, Saitama 350-0495, Japan

[3]Division of Molecular and Cellular Medicine, National Cancer Center Research Institute, 5-1-1 Tsukiji, Chuo-ku, Tokyo 104-0045, Japan

[4]Department of Virology and Liver Unit, Nagoya City University Graduate School of Medical Sciences, 1 Kawasumi, Mizuho-ku, Nagoya, 467-8601, Japan

[$]These authors contributed equally to this work

[*]Corresponding authors: Dr. Yutaka Enomoto, Laboratory of Cell Growth and Differentiation, Institute of Molecular and Cellular Biosciences, The University of Tokyo; 1-1-1 Yayoi, Bunkyo-ku, Tokyo, 113-0032, Japan; Phone: (+81-3) 5841-7884; Fax: (+81-3) 5841-8475 (email: yenomoto@iam.u-tokyo.ac.jp)

Additional Correspondence: Dr Atsushi Miyajima, Laboratory of Cell Growth and Differentiation, Institute of Molecular and Cellular Biosciences, The University of Tokyo; 1-1-1 Yayoi, Bunkyo-ku, Tokyo, 113-0032, Japan; Phone: (+81-3) 5841-7884; Fax: (+81-3) 5841-8475 (email: miyajima@iam.u-tokyo.ac.jp)

Figure S1. Extracellular HBV DNA secretion in HBV-infected PHHs.

Cultured media were collected from PHHs on day 3 and 7 after HBV infection. Extracellular HBV DNA was extracted from media and then examined by real-time quantitative PCR.

Figure S2. Long 3' UTR of human IL-21 mRNA was identified from another donor T cells.

(**A**) RT-PCR was performed with RNA of human Th2 cells derived from another donor to detect human IL-21 mRNA. Revere primers were designed in putative regions of 3' UTR of human IL-21 mRNA. One representative data of three experiments is shown. (**B**) The 3' end of human IL-21 mRNA was identified by 3' RACE. The forward primer was designed to anneal to the 2463-2484 nt of the 3' UTR. Asterisks indicate non-specific bands.

Figure S3. Human IL-21 mRNA with the long 3' UTR is a major transcript.

(**A**) Black bars indicate the regions amplified by quantitative real-time RT-PCR. (**B**) Total RNAs derived from human Th1 cells were treated with Deoxyribonuclease I (Invitrogen), and reverse transcribed by using SuperScript VILO cDNA Synthesis Kit (ThermoFisher). Quantitative real-time RT-PCR was performed with primers as follows: 5′-aggaaaccaccttccacaaa-3′ and 5′ -gaatcacatgaagggcatgtt-3′ for A1 region and 5′ -tgagtctgatgttcagttgctcagt -3′ and 5′- ccttttctcccctccttcctt -3′ for A2 region. Data are shown as

means ± SEM of three independent experiments performed. Fold change indicates the efficiency to the power of the Cq.

Figure S4. Full-length 3’ UTR of human IL-21 mRNA.

Identified 2602 nucleotides of the long 3’UTR of human IL-21 cDNA were shown. Conserved miRNA binding sites are highlighted in light blue. Major poly A signal sequences, AAUAAA or AUUAAA, are highlighted in green.

Figure S5. miRNA expressions in EVs derived from HBV-infected PHHs.

Relative expression levels of EV-miRNAs were examined by quantitative RT-PCR. The expression levels of each miRNA were normalized by total RNA amount of EVs. Bars indicate the means of triplicates.

Figure S6. Reporter vectors with mutant 3’ UTR of human IL-21.

Reporter vectors with mutant 3’ UTR of human IL-21 are shown. Mutations are generated on the coplementary site for the seed region of each miRNA.

Figure S7. Transduction of miRNAs into human Th2 cells.

Relative expression levels of miRNAs were examined by quantitative real-time RT-PCR at 24 hours after the transfection. The expression levels were normalized to U6. Data are shown

as means + SEM of three independent experiments performed. Statistical significance was determined by one-tailed Welch's t test. * $P< 0.05$, ** $P< 0.01$.

Figure S8. Effect of Entecavir on miRNA expressions in EVs derived from HepG2.2.15.7 cells.

Relative expression levels of EV-miRNAs in HepG2.2,15,7 cells with 0 - 3 nM Entecavir were examined by quantitative RT-PCR. The expression levels of each miRNA were normalized by total RNA amount of EVs. Bars indicate the means of triplicates.

Figure S1

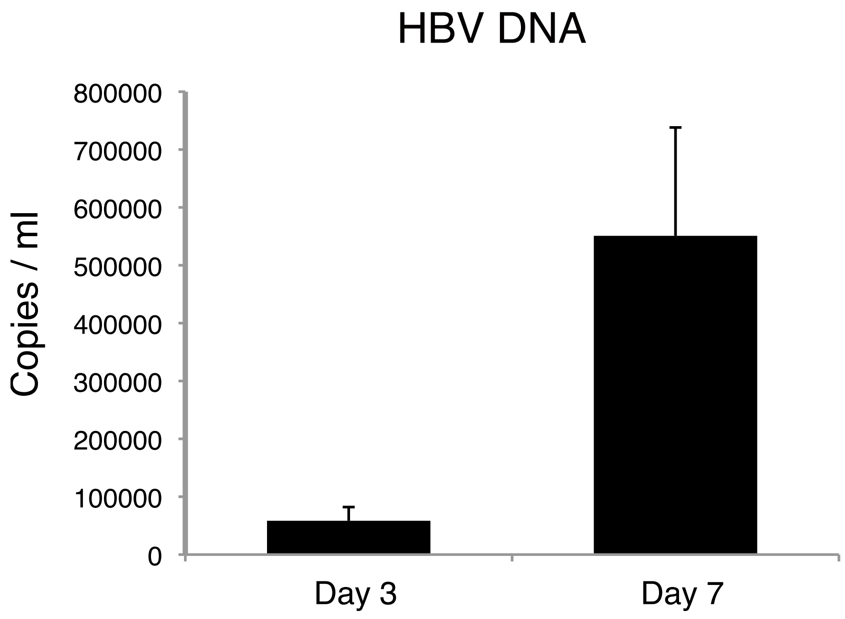
HBV DNA
Copies / ml
800000
700000
600000
500000
400000
300000
200000
100000
0
Day 3
Day 7

Figure S2

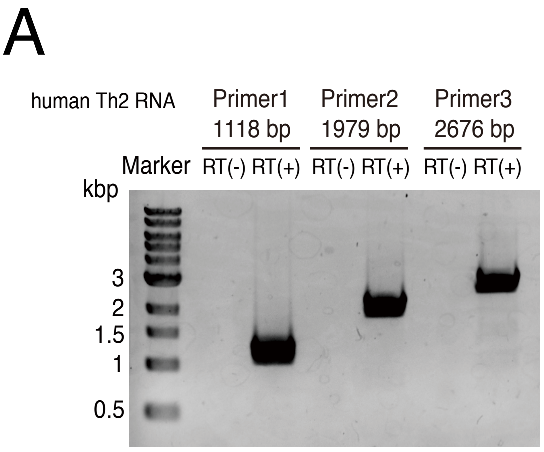
A
human Th2 RNA
Primer1
1118 bp
Primer2
1979 bp
Primer3
2676 bp
Marker
RT(-) RT(+)
RT(-) RT(+)
RT(-) RT(+)
kbp
3
2
1.5
1
0.5

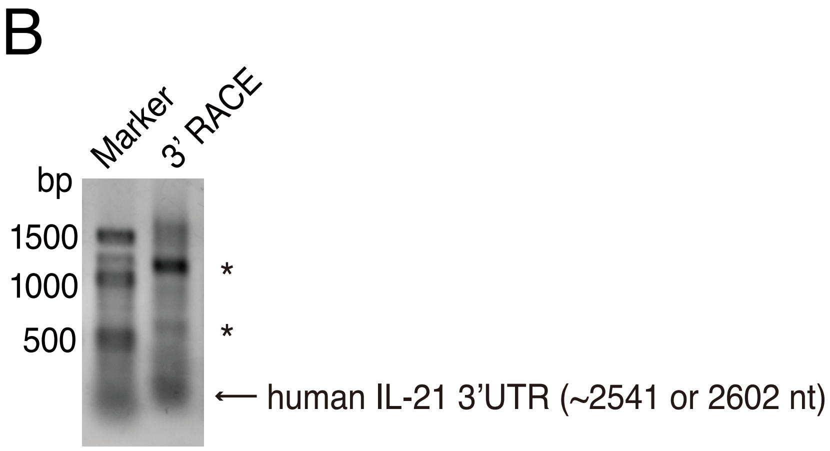
B
Marker
3' RACE
bp
1500
1000
500
*
*
human IL-21 3'UTR (~2541 or 2602 nt)

Figure S3

A

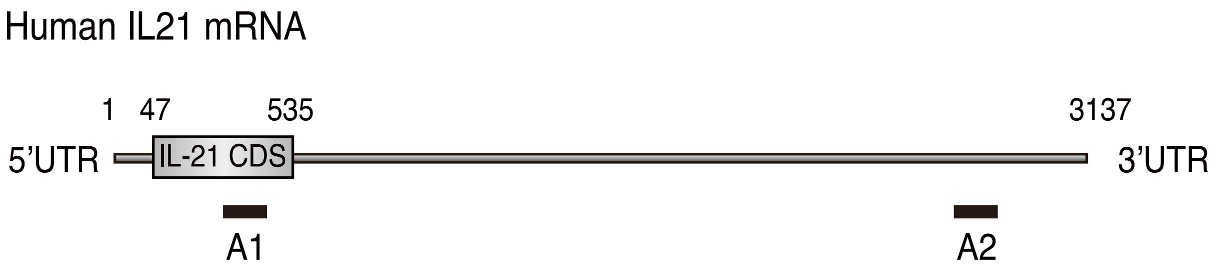

B

Amplified region	Efficiency	Cq	Fold change (×10^7)	Relative ratio
A1: CDS region (359~426 nt)	1.961	25.14±0.087	2.295±0.116	1.000±0.048
A2: 3' UTR (2746~2863 nt)	2.053	23.76±0.017	2.645±0.033	0.864±0.011

Figure S4

GGATCTAACTTGCAGTTGGACACTATGTTACATACTCTAATATAGTAGTGAAAGTCATTTCTTTGTATTCCA
AGTGGAGGAGTACAATATATTAGCGATGGGAAAAAAAAACTCATAAGTGTGCAAAGTCAGGATTATTTCC
CCATAATCACTATACAATAGTCTGATTTCCTATGTTATTGATTTCTATGACTTCTGAAGTTTTTACTTTATCT
CACTGCCCAACTTTGACATTTCTTGGTTGAAGGAGAAGGCTAAAATTTTTAACCAAAAATACAGTTTTGAC
ATCTATTGATTTTATATTAACCTTGAGCTTGATTACCACCAGAATGAGGTCTATTATGATCATAACTTAAAA
CATATATACCTTCATTCCAGCTGATAATATAGAAGCATAGAAGCAAAATATATAAAAGCAAAATGAAAAG
TTGACATCTTTATTCTTTTAAGGAAATGAAAACTGAGTGTTTAAAAACTTATATCTGAATAGTTTTTTAAAT
CTCAGAATGTCTAACATATAAATATGTAATTTATTTATGCACCGGGGGATACTAGATGGCTATAAAAGCCT
TACTTCTTTATTGGGGTTTATAAAAATATTTAAATACTATAGATATGAATGTATTTATGGAATAGACTCTTA
GTCAGTGGCAATAGAGTATTAAGAAGTGGATTTAATATTACCTTCCTCCAGCCCCCAGTCCCCTTATAAGC
TAACATTTTAAGCTTGTGGAAATGGCAATTTAAACCTATAGAGGGACATATGCCAGGTTGTCTTCTGGAAC
CAAACATAGTTTTAAAAGTATTGTAGCTCTCTTCTCAAACTGTTATAATGATCATATTATGAGCAAGCTAG
TAACTCCAAATATTTGTCTCCTTCTTTCTTCTTTCGTTTCTTTTCTTCCTTCTTTTTAAAATGAAACTTCTGCT
TTGACAAAATTTCACAACATGTTTAACATTTTGGACACTTATAGTTACTATACTTTGGATAAGACAAATTC
AATCACCTATATAATTTAAAAATCAGGAGACAAACCAAGAGGCCAGGACTCCTGTCCAAGTTGAAAACAG
ACTTTCGCATAAGCATTCCAATCTTTCAGACTTGGGTAGTAAAAACAAGCAACCTACTCTACCTGCCATTT
CATATCAACTCCAGCACAGCTTGTTTAAACATGAAGTTAAAAACTCTAGAAAAGTAAAATGTTCGCTAGA
ATGGTATAAGCTATTTGAGAATCTCTTATTGTTAAAATAGTTGTGATTATGCAAGAGGATGTGGGATGAGA
AATGCTTTATGCTCTGAGAAGAAAAATTATGTGCTTAAAACAAATACAGACTTAACCTACAGTTCTAAAA
GATATATTGAGAATTATTTAATCAAATCTCAATGAAATGTTTTATCATCAGCAAGTAGACTCAGAAAAAA
GAAAAAAAAATTTATCATCAGAAACAAGGTACTCTGTTTTAACATTTTAGAACATGTATGACAAAGGAAT
TATGTGCCTGATGATGATAATATATATCTAAAATGTGCCTCAGTTTCAAATACGCCAGCCCGGGTTGTTGT
GACAAAATGTGGAAGCAGGCACAAAGATAGTGAGAAAGATGGAATCTGTATTTCTTGTTTCTGAATGTAT
AGCTATTAACTTCTTGGATATTGTATTTCATATTTAAAATAATGTATTTCTTTTGGCATGTTTATTGTTAAAA
ATTTCTGTTCCAATTATTTTGATCAGGGTCTCTATCTTAAAAGTAAAAATAACTCAGTGAGGGTTTGGCTA
AGAGCCTTCATTAAACAGAGTTTAAACACCCAGATAAACTCTGCTGTTTAACCAGAGTTAAACCCACCAG
CACTCCAGGAAAGGCAATGCATTGGAATAAACAAGATGAAGCAGTGAGACTTGAACAGCTCCATCTTCAC
TGCTCTATGTTCAAGATGGCCAGTTCTTAAAAGGAATATAAAGATAACCCATAAATATAACTGTTCATCTA
TGGACAGTAATACAGGAGACGTGAAATTTTGTTGACTATGAAATAGTGGTCACTGTTTAAACTCTTTGGGT
TTCCATTTCTTTATCTGTAGAATGAGAGTGCTGGACTTAATCTCTAATGTCTATTCTTTCTTTTTTAAAGCTT
TAAATGTCTATGAATATCTAAAGTTCTGGGTATTCTTACAGAAACATCTGATCATACTTCTGAGTAAAACT
TTCTACTGAGTCTGATGTTCAGTTGCTCAGTATTATTTCAGTAACATTTGTAGCAACACTCACTGCAGGAAC
AAGGGACATATTCCAAGAAAAAAAAGGAAGGAAGGAAGGAGGGGAGAAAAGGAAACATTATTATAGGT
CATGAGAGTTCAGGGAAAATTAATCATGATCTTTATTATTTTAGTTCAAAGTGTCTTCTTGGAACACTTGTA
TAAAATTGTAGCATTATTATTCTATATTATTGCTCTGTTGTATTTACATATGCATCTGAGAATTTAGCTAAT
ATGAACTATGTAGTTTATAACTTAATAATTATTTATTATATATTTGATTTTAAATGTTCATGTTTATGGCTTC
TTATTTAAGACCTGATCATATTAAATACTACCCGCCCAGTA

Figure S5

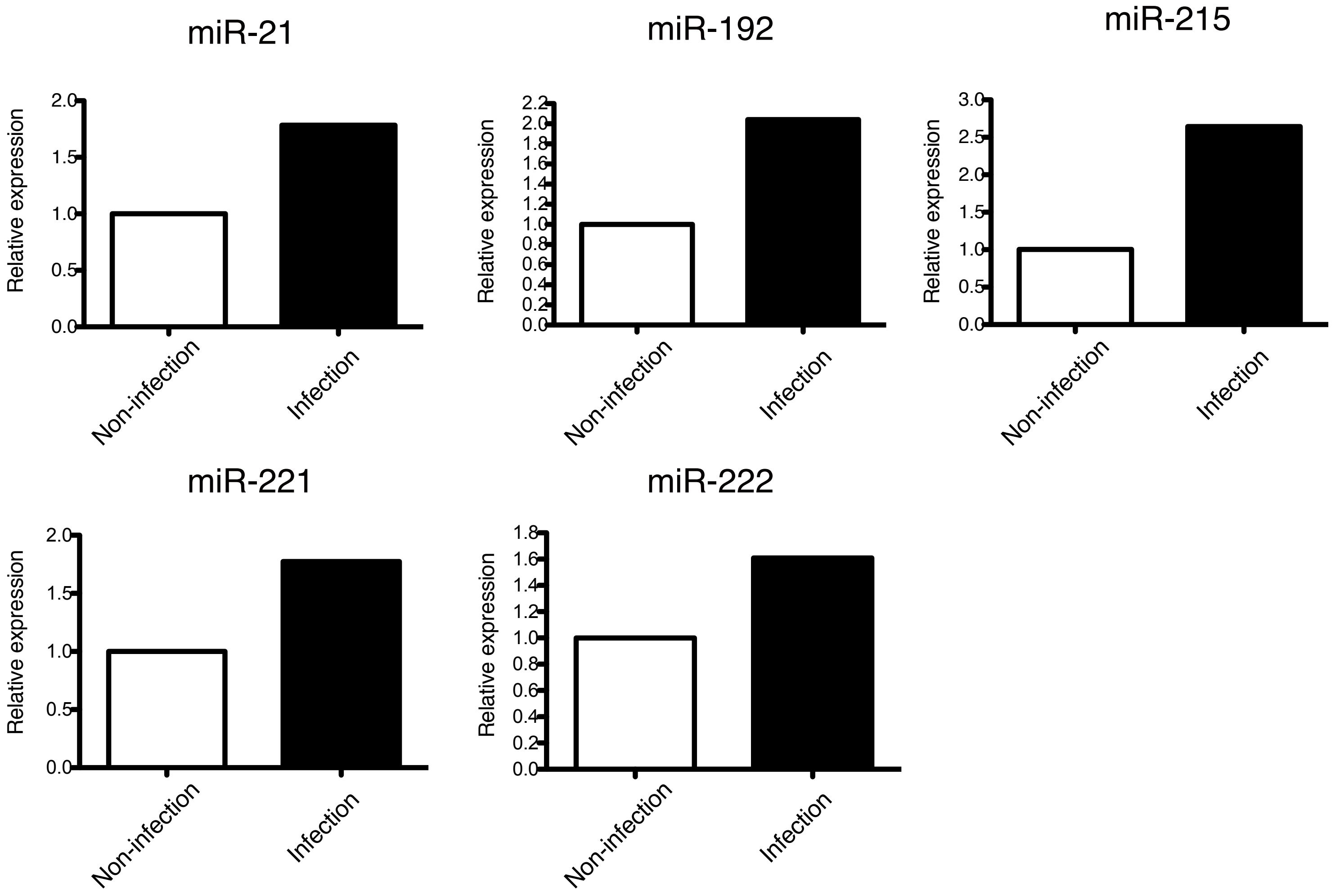

miR-21
Relative expression
2.0
1.5
1.0
0.5
0.0
Non-infection
Infection
miR-192
Relative expression
2.2
2.0
1.8
1.6
1.4
1.2
1.0
0.8
0.6
0.4
0.2
0.0
Non-infection
Infection
miR-215
Relative expression
3.0
2.5
2.0
1.5
1.0
0.5
0.0
Non-infection
Infection
miR-221
Relative expression
2.0
1.5
1.0
0.5
0.0
Non-infection
Infection
miR-222
Relative expression
1.8
1.6
1.4
1.2
1.0
0.8
0.6
0.4
0.2
0.0
Non-infection
Infection

Figure S6

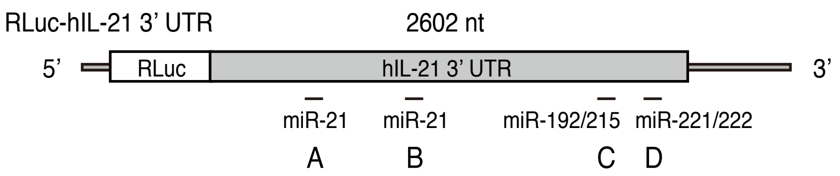

Mut AB

site A
WT 5' ---CAGCCCCCAGUCCCCUUAUAAGCUA---
Mutant 5' ---CAGCCCCCAGUCCCCUUACACGAUA---

site B
WT 5' ---UUCGCUAGAAUGGUAUAAGCUA---
Mutant 5' ---UUCGCUAGAAUGGUACACGAUA---

Mut C

site C
WT 5' ---GGAAACAUUAUUAUAGGUCAU---
Mutant 5' ---GGAAACAUUAUUAUCGAUGAU---

Mut D

site D
WT 5' ---AACACUUGUAUAAAAUUGUAGCA---
Mutant 5' ---AACACUUGUAUAAAAUCGCAACA---

Figure S7

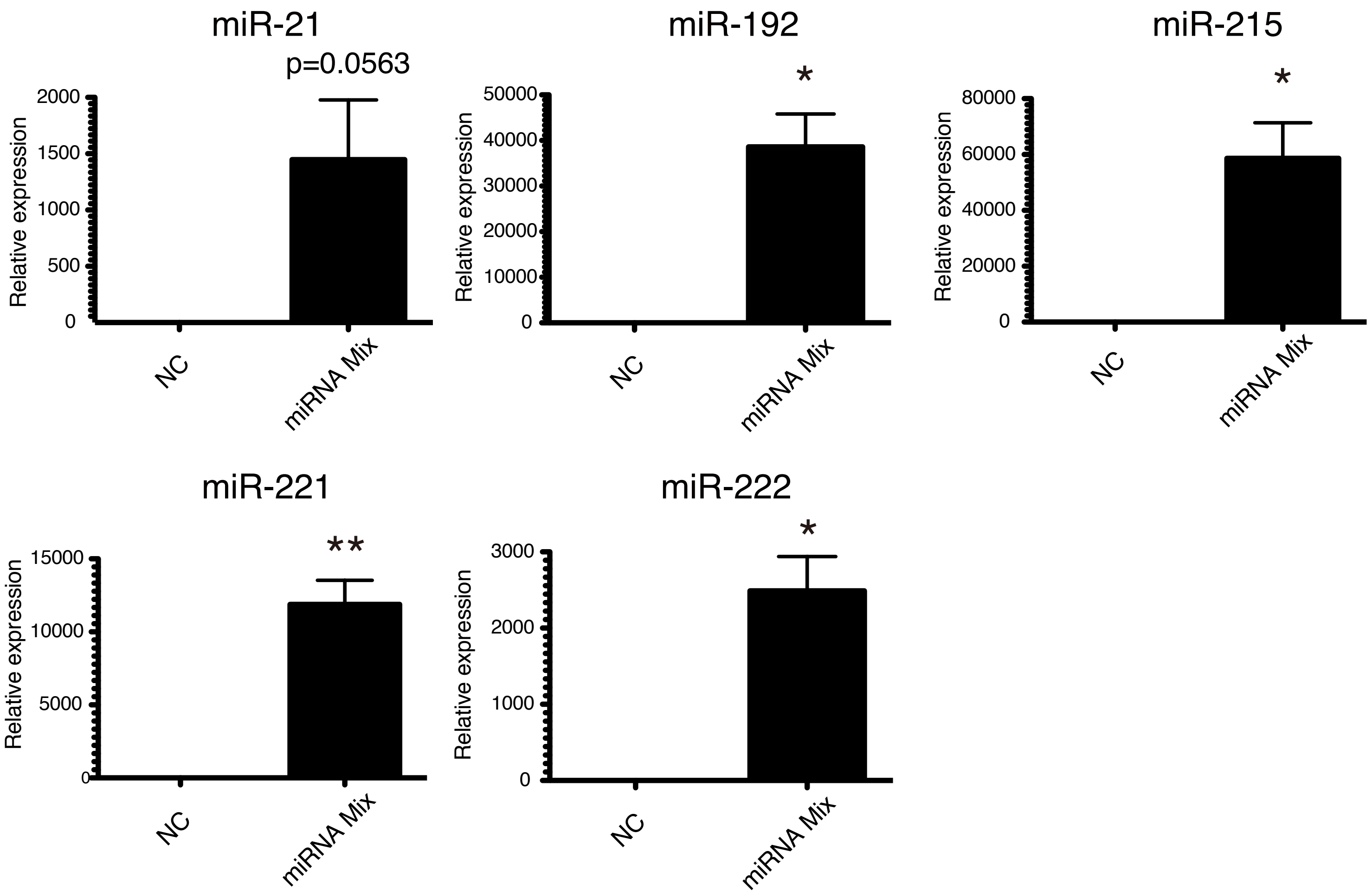
miR-21
p=0.0563
Relative expression
2000
1500
1000
500
0
NC
miRNA Mix
miR-192
*
Relative expression
50000
40000
30000
20000
10000
0
NC
miRNA Mix
miR-215
*
Relative expression
80000
60000
40000
20000
0
NC
miRNA Mix
miR-221
**
Relative expression
15000
10000
5000
0
NC
miRNA Mix
miR-222
*
Relative expression
3000
2000
1000
0
NC
miRNA Mix

Figure S8

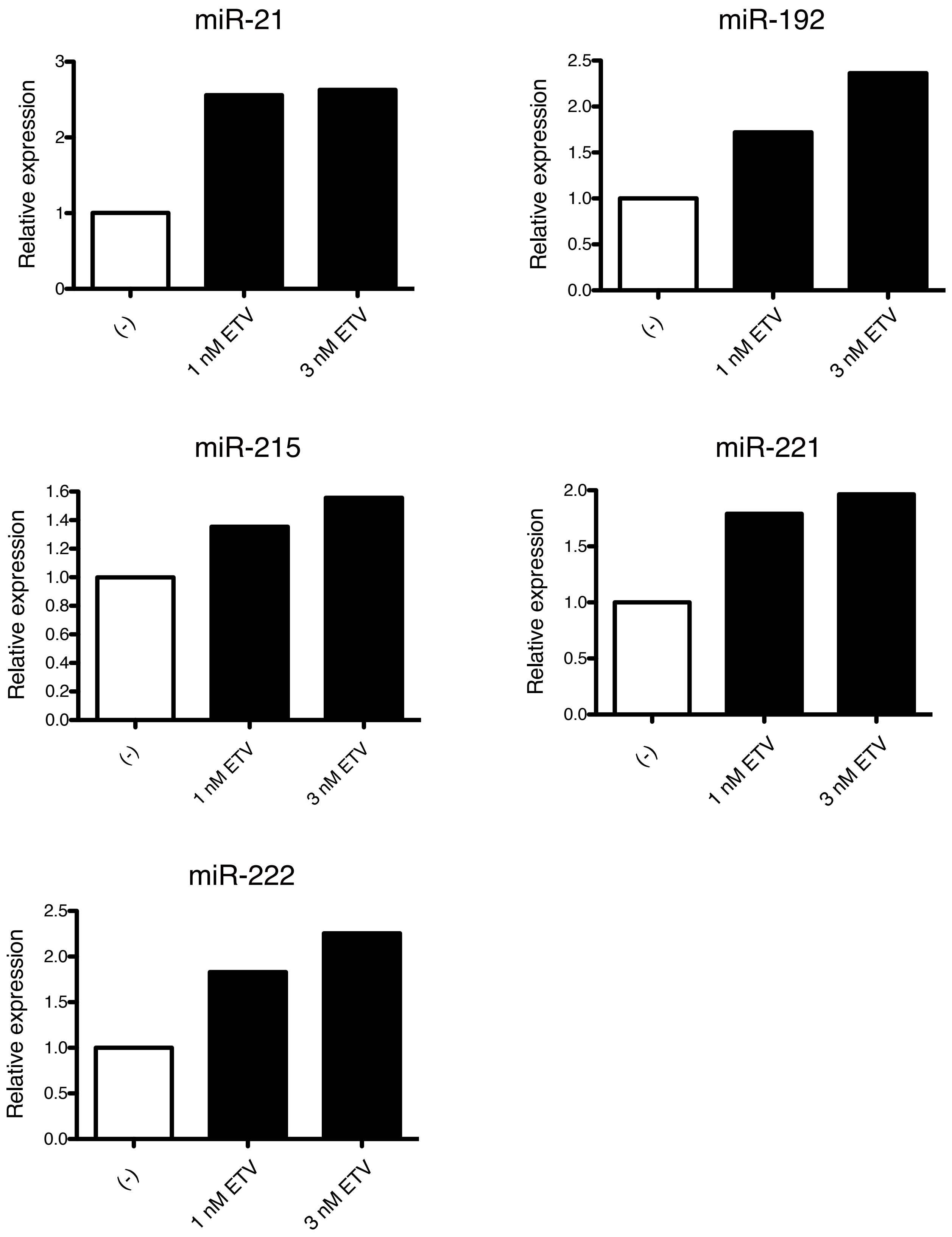
miR-21
miR-192
miR-215
miR-221
miR-222
Relative expression
(-)
1 nM ETV
3 nM ETV

Table S1. Microarray analysis of EV-miRNAs with HBV infection.

Name	miRbase Accession No.	Infection	Non-infection	Fold change
hsa-miR-378d	MIMAT0018926	125.2	21.8	5.73
hsa-miR-3678-3p	MIMAT0018103	91.3	21.1	4.33
hsa-miR-411*	MIMAT0004813	97.0	22.9	4.23
hsa-miR-30e	MIMAT0000692	324.7	77.0	4.22
hsa-miR-106a	MIMAT0000103	597.0	143.6	4.16
hsa-miR-378e	MIMAT0018927	98.1	23.7	4.14
hsa-miR-16	MIMAT0000069	709.4	176.1	4.03
hsa-miR-221	MIMAT0000278	156.2	39.5	3.96
hsa-miR-3687	MIMAT0018115	138.6	36.3	3.82
hsa-miR-215	MIMAT0000272	498.5	132.1	3.77
hsa-miR-19b	MIMAT0000074	934.6	247.8	3.77
hsa-miR-378i	MIMAT0019074	142.8	39.2	3.64
hsa-miR-21	MIMAT0000076	2021.7	561.5	3.60
hsa-miR-192	MIMAT0000222	1756.9	489.8	3.59
hsa-miR-27b	MIMAT0000419	1161.7	326.0	3.56
hsa-miR-513a-5p	MIMAT0002877	920.4	261.8	3.52
hsa-miR-658	MIMAT0003336	1043.9	300.2	3.48
hsa-miR-320e	MIMAT0015072	182.1	52.7	3.46
hsa-miR-34a	MIMAT0000255	210.6	63.4	3.32
hsa-miR-148a	MIMAT0000243	608.5	183.9	3.31
hsa-miR-494	MIMAT0002816	708.5	216.0	3.28
hsa-miR-30b	MIMAT0000420	734.3	224.6	3.27
hsa-miR-222	MIMAT0000279	113.5	34.9	3.26
hsa-let-7c	MIMAT0000064	907.0	279.2	3.25
hsa-miR-4792	MIMAT0019964	603.2	188.3	3.20
hsa-miR-27a	MIMAT0000084	612.3	192.2	3.19
hsa-miR-99a	MIMAT0000097	441.0	139.0	3.17
hsa-miR-17	MIMAT0000070	459.8	147.8	3.11
hsa-miR-22	MIMAT0000077	2683.4	863.5	3.11
hsa-miR-20a	MIMAT0000075	388.1	125.6	3.09
hsa-miR-320d	MIMAT0006764	144.8	47.1	3.07
hsa-miR-122	MIMAT0000421	13282.7	4372.0	3.04
hsa-miR-30a	MIMAT0000087	379.0	125.7	3.02
hsa-miR-320b	MIMAT0005792	194.0	64.5	3.01
hsa-let-7i	MIMAT0000415	86.5	28.8	3.00
hsa-miR-29a	MIMAT0000086	769.2	259.1	2.97
hsa-miR-15a	MIMAT0000068	290.8	99.2	2.93
hsa-miR-107	MIMAT0000104	299.6	103.9	2.88
hsa-miR-26a	MIMAT0000082	1062.5	369.4	2.88
hsa-let-7d	MIMAT0000065	784.8	273.1	2.87
hsa-miR-30d	MIMAT0000245	626.1	218.2	2.87
hsa-miR-152	MIMAT0000438	61.0	21.3	2.86
hsa-miR-4632	MIMAT0019688	63.4	22.3	2.84
hsa-miR-151b	MIMAT0010214	91.5	32.4	2.82
hsa-miR-92b	MIMAT0003218	379.4	134.8	2.81
hsa-miR-92a	MIMAT0000092	628.2	223.7	2.81
hsa-miR-378c	MIMAT0016847	155.9	56.1	2.78
hsa-miR-3679-5p	MIMAT0018104	1422.2	520.9	2.73
hsa-miR-100	MIMAT0000098	588.9	215.8	2.73
hsa-miR-24	MIMAT0000080	2574.7	952.1	2.70
hsa-miR-101	MIMAT0000099	58.7	21.9	2.68
hsa-miR-193a-3p	MIMAT0000459	78.4	29.3	2.68
hsa-miR-191	MIMAT0000440	337.9	126.5	2.67
hsa-miR-2113	MIMAT0009206	99.6	37.3	2.67
hsa-miR-135a*	MIMAT0004595	57.8	21.8	2.65
hsa-miR-29b	MIMAT0000100	221.9	85.1	2.61
hsa-miR-26b	MIMAT0000083	438.1	169.2	2.59

hsa-miR-4459	MIMAT0018981	2511.8	981.6	2.56
hsa-miR-106b	MIMAT0000680	125.7	49.2	2.55
hsa-miR-125b	MIMAT0000423	609.5	240.4	2.53
hsa-miR-422a	MIMAT0001339	139.7	55.1	2.53
hsa-miR-3135b	MIMAT0018985	2731.0	1078.7	2.53
hsa-miR-30c	MIMAT0000244	805.3	322.9	2.49
hsa-miR-23a	MIMAT0000078	947.9	380.1	2.49
hsa-miR-320c	MIMAT0005793	159.5	64.0	2.49
hsa-miR-4638-5p	MIMAT0019695	1231.4	496.3	2.48
hsa-let-7a	MIMAT0000062	912.4	367.8	2.48
hsa-miR-15b	MIMAT0000417	133.4	54.3	2.46
hsa-miR-103a	MIMAT0000101	342.1	139.5	2.45
hsa-miR-3714	MIMAT0018165	418.0	170.9	2.45
hsa-miR-4324	MIMAT0016876	257.1	105.2	2.44
hsa-miR-4476	MIMAT0019003	898.8	368.1	2.44
hsa-miR-451	MIMAT0001631	108.2	44.8	2.42
hsa-miR-185	MIMAT0000455	51.1	21.2	2.41
hsa-miR-23a*	MIMAT0004496	68.7	28.6	2.40
hsa-miR-20b	MIMAT0001413	282.3	117.5	2.40
hsa-miR-4741	MIMAT0019871	1553.6	647.8	2.40
hsa-miR-194	MIMAT0000460	1222.0	510.6	2.39
hsa-let-7b	MIMAT0000063	588.1	252.2	2.33
hsa-miR-214	MIMAT0000271	123.8	53.4	2.32
hsa-miR-3616-3p	MIMAT0017996	814.7	355.8	2.29
hsa-miR-4481	MIMAT0019015	83.4	36.9	2.26
hsa-miR-19a	MIMAT0000073	132.4	58.9	2.25
hsa-miR-23b	MIMAT0000418	1283.1	571.4	2.25
hsa-miR-365*	MIMAT0009199	2242.5	999.4	2.24
hsa-miR-126	MIMAT0000445	203.3	92.3	2.20
hsa-miR-2392	MIMAT0019043	340.4	154.5	2.20
hsa-miR-29c	MIMAT0000681	71.3	32.6	2.19
hsa-miR-1307	MIMAT0005951	433.5	200.5	2.16
hsa-miR-198	MIMAT0000228	100.4	46.6	2.15
hsa-miR-1908	MIMAT0007881	30516.0	14289.2	2.14
hsa-miR-1973	MIMAT0009448	60.5	28.4	2.13
hsa-miR-1203	MIMAT0005866	57.8	27.2	2.12
hsa-miR-4484	MIMAT0019018	17541.8	8271.4	2.12
hsa-miR-4257	MIMAT0016878	1669.2	791.7	2.11
hsa-miR-4787-5p	MIMAT0019956	39264.1	18748.0	2.09
hsa-miR-4749-5p	MIMAT0019885	5361.0	2582.4	2.08
hsa-miR-4505	MIMAT0019041	2678.0	1293.3	2.07
hsa-miR-122*	MIMAT0004590	424.3	205.9	2.06
hsa-miR-93	MIMAT0000093	123.6	60.0	2.06
hsa-miR-130a	MIMAT0000425	116.4	56.6	2.05
hsa-miR-125a-5p	MIMAT0000443	137.9	67.6	2.04
hsa-miR-921	MIMAT0004971	176.4	86.5	2.04
hsa-miR-4467	MIMAT0018994	26648.6	13076.4	2.04

Table S2. Expression levels of EV-miRNAs with HBV infection at day 2.

Name	miRbase Accession No.	Infection	Non-infection	Fold change
hsa-miR-21	MIMAT0000076	1822.5	768.9	2.37
hsa-miR-192	MIMAT0000222	1843.7	1079.7	1.71
hsa-miR-215	MIMAT0000272	424.1	287.2	1.48
hsa-miR-221	MIMAT0000278	122.6	76.1	1.61
hsa-miR-222	MIMAT0000279	88.2	41.5	2.13
